# On the alleged origin of geminiviruses from extrachromosomal DNAs of phytoplasmas

**DOI:** 10.1186/1471-2148-11-185

**Published:** 2011-06-28

**Authors:** Federica Saccardo, Emanuele Cettul, Sabrina Palmano, Emanuela Noris, Giuseppe Firrao

**Affiliations:** 1 Dipartimento di Biologia e Protezione delle Piante, Università di Udine, via Scienze 208, 33100 Udine, Italy; 2 Istituto di Virologia Vegetale, CNR, Strada delle Cacce 73, 10135 Torino, Italy; 3 Istituto Nazionale di Biostrutture e Biosistemi, Interuniversity Consortium, Italy

## Abstract

**Background:**

Several phytoplasmas, wall-less phloem limited plant pathogenic bacteria, have been shown to contain extrachromosomal DNA (EcDNA) molecules encoding a replication associated protein (Rep) similar to that of geminiviruses, a major group of single stranded (ss) DNA plant viruses. On the basis of that observation and of structural similarities between the capsid proteins of geminiviruses and the *Satellite tobacco necrosis virus*, it has been recently proposed that geminiviruses evolved from phytoplasmal EcDNAs by acquiring a capsid protein coding gene from a co-invading plant RNA virus.

**Results:**

Here we show that this hypothesis has to be rejected because (i) the EcDNA encoded Rep is not of phytoplasmal origin but has been acquired by phytoplasmas through horizontal transfer from a geminivirus or its ancestor; and (ii) the evolution of geminivirus capsid protein in land plants implies missing links, while the analysis of metagenomic data suggests an alternative scenario implying a more ancient evolution in marine environments.

**Conclusion:**

The hypothesis of geminiviruses evolving in plants from DNA molecules of phytoplasma origin contrasts with other findings. An alternative scenario concerning the origin and spread of Rep coding phytoplasmal EcDNA is presented and its implications on the epidemiology of phytoplasmas are discussed.

## Background

Geminiviruses are a large group of plant viruses causing several important diseases worldwide, characterized by a nucleic acid genome encapsidated into twinned particles formed by joining two incomplete icosahedra. Geminiviruses differ from most other plant viruses in the fact that they are single-stranded DNA (ssDNA) viruses that multiply through rolling circle replication (RCR). They constitute one of the three recognized groups of episomal replicons that use RCR, the other being circular ssDNA bacteriophages, and plasmids of bacteria or archaea [1]. In a seminal paper Koonin and Ilyina [2] found weak similarities between the replication associated protein (Rep) of geminiviruses and that of the pLS1 family of plasmids of Gram positive bacteria. Despite the limited similarity, the conservation of motif signatures and of the spacing between them led to the conclusion that they constitute a distinct superfamily. On this basis Koonin and Ilyina [2] advanced the hypothesis that geminiviruses may have actually originated from bacterial plasmids.

In the late 1990s, sequences with a relatively high similarity to Rep were found in some extrachromosomal DNA molecules (EcDNA) borne by a group of phytoplasmas related to the Western-X disease phytoplasma [3], and then in the EcDNAs of several other phytoplasmas [4-9]. Phytoplasmas are plant pathogenic Mollicutes, wall-less prokaryotes taxonomically related to the *Clostridium/Bacillus *clade of low G+C Gram positive bacteria. They share with geminiviruses the characteristic of inhabiting the plant phloem and being transmitted from plant to plant by defined groups of insect vectors. The similarity of replication associated protein of phytoplasma EcDNAs and geminiviruses has been a matter for discussion among plant pathologists over the last ten years [10,11].

On the basis of similarities among replication associated proteins and comparative homology-based structural modeling of viral capsid proteins, Krupovic and coworkers [12] recently proposed "a plasmid-to-virus transition scenario, where a phytoplasmal plasmid acquired a capsid-coding gene from a plant RNA virus to give rise to the ancestor of geminiviruses". Here we report some new experimental data, homology searches and phylogenetic analysis that, together with the results of previous research, conclusively show that this, although fascinating, hypothesis is too simplistic and other possible scenarios are more likely.

## Methods

### Plant sources

Phytoplasma strains were maintained in a greenhouse by graft-transmission to healthy *Catharanthus roseus*. The phytoplasma strains used in this work and their origin are listed in Additional File 1. Nucleic acids from healthy and infected periwinkle plants were isolated using a standard phytoplasma enrichment procedure [13].

### DNA/Protein sequence sources and analysis

The sequence data used in this work relative to 16S rDNA and single stranded DNA binding (SSB) proteins of various bacteria, plasmid replication protein (rep), phytoplasmal EcDNAs, virus capsid and replication associated proteins, as well as environmental DNA were retrieved from the EMBL database and the community cyberinfrastructure for advanced marine microbial ecology research and analysis (CAMERA, http://camera.calit2.net). The complete EcDNA sequence of New Jersey Aster Yellows (NJAY) phytoplasma was determined in this study. Sequence accessions, genes, organism names, reference databases and labels used in the figures are listed in Additional File 2.

Multiple sequence alignments of 16S rRNA genes, rep and SSB were performed separately using MEGA4 [14]. For rep, the helicase domain was excluded and the alignment was restricted to the replication initiator domain (N-terminal region of about 150-180 aa).

Phylogenetic analysis using parsimony was carried out with the PHYLIP package using the programs SEQBOOT, PROTPAR, DNAPARS and CONSENSE[15]. Bootstrapping with 500 replicates was performed to estimate the stability and support for the interfered clades.

Percent identity and similarity of phytoplasmal EcDNA borne proteins and capsid proteins with other database accessions were calculated using NEEDLE[16], launched recursively with a BIOPERL script when needed. Principal coordinates analysis was carried out with R [17]. The likelihood-ratio test for monophyly [18] was carried out with a selection of 14 sequences taking a null hypothesis that the Rep of type II EcDNAs, the rep of type I EcDNA and RCR plasmids are a group while the Rep of geminiviruses are another. Likelihoods were estimated with PHANGRON[19]. The significance of the likelihood ratio was estimated by parametric bootstrap according to [18] by simulation of 1000 replicated datasets generated with INDEL-SEQ-GEN[20]. Tetranucleotide usage patterns were compared with the program TETRA[21].

### NJAY phytoplasma EcDNA amplification and sequence analysis

Degenerate primer sets (Additional File 3) were designed on conserved EcDNA regions deduced from sequences available from the EMBL database, to PCR amplify the replication associated protein of the EcDNA of "*Candidatus *Phytoplasma asteris" strain NJAY. Purified PCR products were sequenced and the entire EcDNA of NJAY phytoplasma was sequenced by primer walking using newly designed primers (see Additional File 3).

Amplifications were performed in a 20-μl PCR reaction containing 100 ng of template DNA, 200 μM dNTPs, 1 μM of each primer, 1 U of 5 PRIME DNA polymerase with the recommended PCR buffer containing MgCl2 (5 PRIME, Hamburg, Germany). PCR was carried out with an automated thermal cycler (T-Professional Basic, Biometra, Germany). The reactions included an initial denaturation cycle at 94°C for 2 min, then 30 cycles of 94°C for 20 sec, 53°C for 20 sec and 72°C for 3 min. At the end, the reaction mixtures were incubated at 72°C for 10 min and then stored at 4°C.

The DNA fragments were sequenced by standard methods and assembled manually using BIOEDIT 7.0.0 (Tom Hall, Carlsbad, CA, USA). Open reading frames were predicted using ORF FINDER (NCBI, http://www.ncbi.nlm.nih.gov/gorf/gorf.html), using the standard genetic code. Homologous sequences were identified from the GenBank database using the BLASTX programme (http://www.ncbi.nlm.nih.gov/blast/Blast.cgi).

## Results and Discussion

### The origin of the phytoplasmal *Rep *is not bacterial

**Figure 1:**
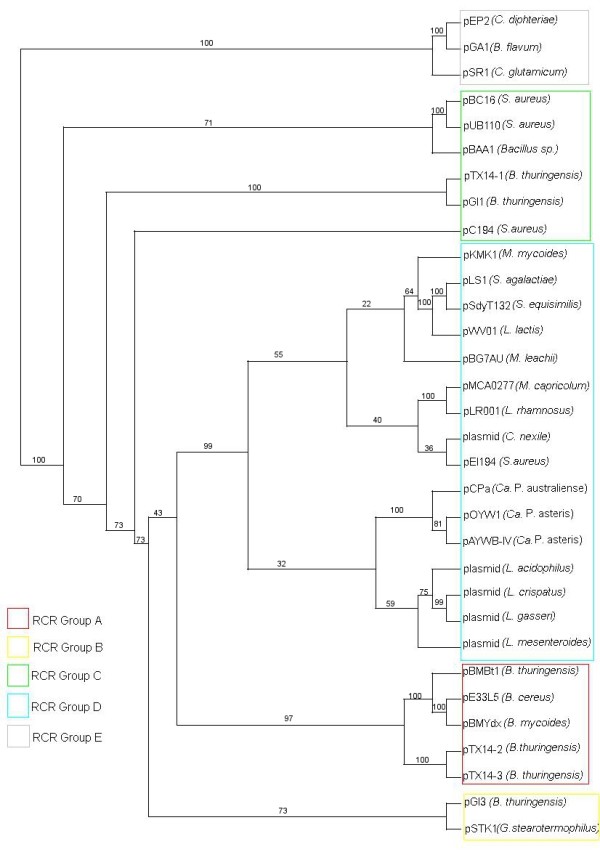
**Phylogenetic tree of RCR Rep proteins**. Phylogenetic analysis of Reps from phytoplasmal type I EcDNA and representatives of different plasmid families of RCR plasmids. Each cluster label letter corresponds to a family as in [52].

During the last 20 years, studies on phytoplasmal DNA showed that there are 3 types of phytoplasmal EcDNAs, according to DNA sequence similarity analysis. While in the most recently discovered type of EcDNAs replication is initiated by a DNA primase encoded by *dnaG*, type I and type II EcDNAs replicate through an RCR mechanism assisted by an EcDNA encoded replication associate protein. Type I molecules include a gene encoding a protein that is phylogenetically related to the replication associated protein (rep) of RCR plasmids of the pLS1 family [22]. Plasmids of this family (PFAM accession: PF01719) have been found in a wide range of Gram positive bacteria, including members of the class Mollicutes. Phytoplasma plasmids differ from other plasmids of the pLS1 family in having a C-terminal region (100 aa) that was related to the reps of circoviruses and the helicases of picorna-like viruses [23]. According to the analysis carried out by Gibbs and coworkers [24] this feature is shared with rep encoded by genes belonging to other RCR bacterial plasmids or integrated into the genome of various organisms, such as *Entamoeba histolytica *and *Lactobacillus acidophilus*. A phylogenetic analysis of the replication associated domain of reps of representatives of the known RCR plasmid families (Figure 1) shows that sequences from different "*Candidatus *Phytoplasma" species are related among themselves and also with sequences from organisms belonging to the low GC branch of Gram positives bacteria, forming a distinct branch of the pLS1 family.

**Figure 2:**
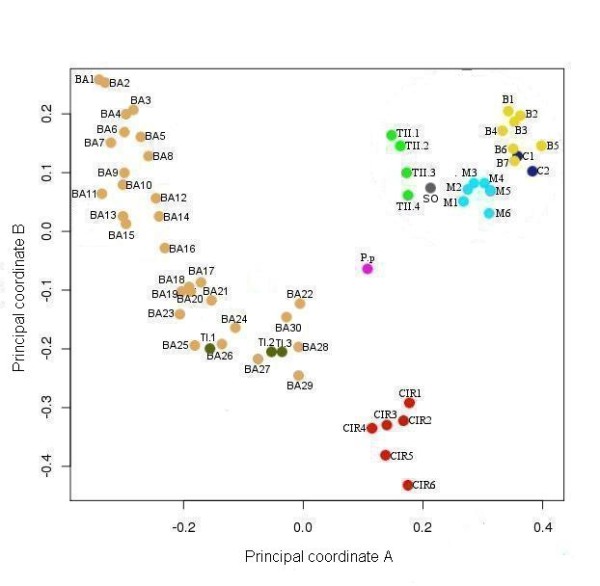
**Analysis of RCR Rep proteins**. Principal coordinate analysis of the distances between RCR replicons of superfamily II (according to [2]) estimated from pairwise similarity of replication associated proteins. Pale-brown dots (BA labelled) represent sequences of bacterial plasmid, red dots (CIR) circoviruses, pale blue dots (M) mastreviruses, yellow dots (B) begomoviruses, violet dots (C) curtoviruses, green-brown dots (TI) phytoplasmal type I EcDNA, bright-green dots (TII) phytoplasmal type II EcDNA, dark blue dots (Ss) SsHSDV-1 and purple (Pp) from *Porphyra pulchra*. See additional file 2 for the detailed explanation of sequence labels.

**Figure 3:**
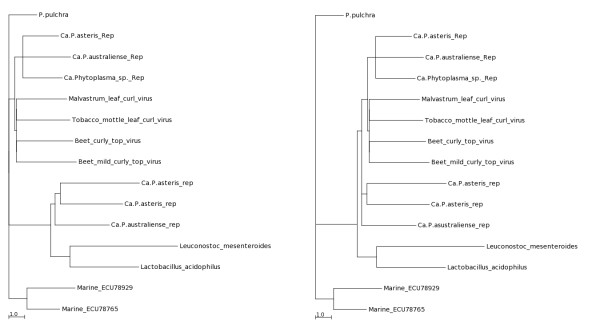
**Evolutionary trees compared with log likelihood ratio**. A: unconstrained tree. B: null hypothesis tree.

Despite the fact that type II EcDNA also replicates through a RCR mechanism [4,25], they encode a protein which is not related to the rep of pLS1, but rather to geminivirus replication associated protein Rep (PFAM accession: PF00799). As noticed earlier, replication associated proteins of viral RCR replicons have no significant similarity with those encoded by plasmid RCR replicons and, as shown in the principal coordinates plot of the pairwise distances of Figure 2, they are a well distinct group of proteins. The phytoplasmal Rep are within the group of viral replicons in Figure 2 as they share high similarity with viral Rep and low similarity with plasmid rep. While there is a high degree of conservation among the replication associated proteins of the same EcDNA type, the rep of type I EcDNA and the Rep of type II EcDNA share modest sequence similarity. To provide statistical evidence that the Rep of type II EcDNA are not phylogenetically related with the rep of the type I EcDNAs (the true plasmids of the phytoplasmas), we carried out a test for monophyly [18] that evaluated by parametric bootstrap the significance of the likelihood ratio of a null hypothesis with the constraint that Rep and rep are monophyletic relative to the unconstrained maximum likelihood tree (Figure 3). The log likelihoods of the null hypothesis and the unconstrained tree resulted -11327.01 and -11264.55, respectively and their ratio (delta = 124.9270) was compared with the delta distribution in a set of alignments of simulated sequences evolved *in silico *using the unconstrained tree as guidance. The largest delta of a set of 500 alignments was 68.13182 and therefore the null hypothesis is to be rejected (P << 0.002). According to a published phylogenetic analysis of phytoplasmal Rep that placed them as a distinct group within the geminivirus Rep clade [12] and due to the failure to find any ancestor or relative for *Rep *among bacterial sequences, we conclude that the Rep of type II EcDNA of phytoplasmas are viral and not bacterial sequences, despite the fact that they have been found associated with bacterial organisms.

### What then are type II EcDNA of phytoplasmas?

**Figure 4:**
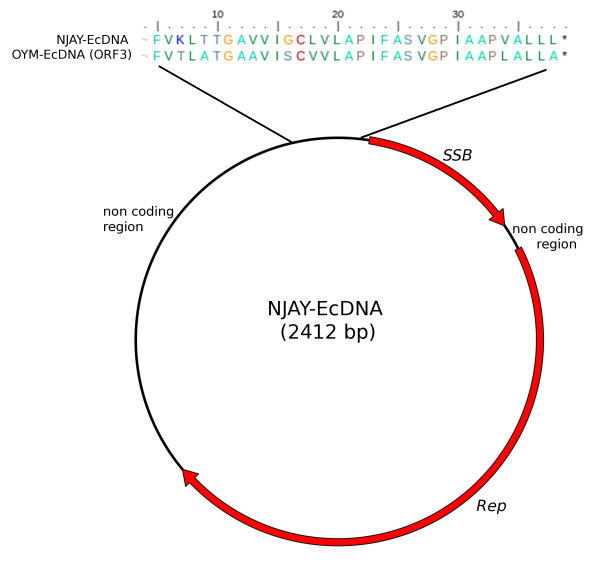
**Schematic structure of the NJAY phytoplasma EcDNA sequenced in this study**. The first nucleotide of Rep is designated as position 1. The arrows indicate the putative ORFs and their direction of transcription. The DNA region corresponding to a remnant part of ORF3 in the non coding region is delimited and expanded on the top of the figure showing the potential translated sequence aligned to part of ORF3 in the EcDNA of the Onion Yellows phytoplasma (accession AB479514.1).

**Figure 5:**
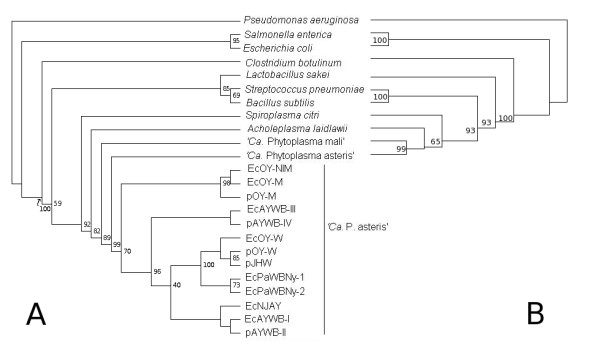
**Maximum likelihood trees constructed by parsimony analysis of SSB proteins (A) and 16S rRNA genes (B) of various Gram positive bacteria and phytoplasmas**. See Additional File 2 for further information on labels. Numbers at nodes are percent bootstrap support values.

**Figure 6:**
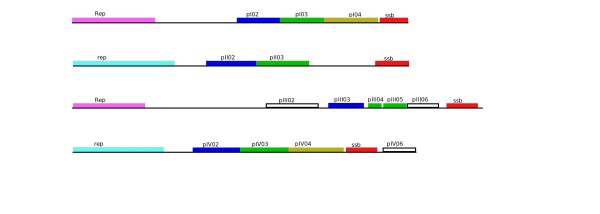
**Gene organization in the four EcDNAs (AYWB-pI, AYWB-pII, AYWB-pIII, AYWB-pIV) of "*Candidatus *Phytoplasma asteris" strain AYWB**. Genes with the same colour share more than 60% similarity in their putatively coded protein. EcDNA sequences were obtained in [4].

**Figure 7:**
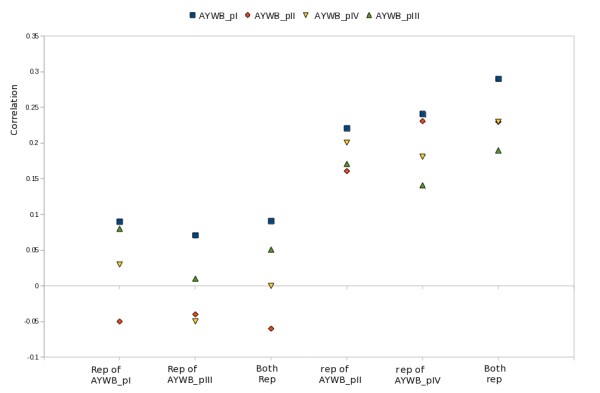
**Correlation between the tetranucleotide patterns used in *rep *and Rep genes of AYWB phytoplasma EcDNAs and the tetranucleotide patterns used in other proteins of the same EcDNAs**.

In order to clarify the origin of type II EcDNAs, we investigated the additional sequences that are part of these replicons. By reviewing the results of Southern blot analyses carried out in our laboratories on DNA extracted from our collection of phytoplasmas using *Rep *sequences as probes, we identified a minimal-sized type II EcDNA in "*Ca*. P. asteris", strain New Jersey Aster Yellows. This 2,400 bp-long EcDNA was cloned and sequenced and was shown to include a *Rep *gene, a gene encoding a ssDNA binding protein (SSB) and a non coding region about 900 bp in length (Figure 4). Database analysis confirmed that a gene for a SSB protein is encoded by all type I and type II phytoplasma EcDNAs sequenced so far, with the exception of three EcDNAs of "*Candidatus *Phytoplasma australiense" (that however has some putative chromosome encoded phage derived SSBs) and two EcDNAs that were isolated from strains that contain multiple different EcDNAs. It is well assessed that RCR replication needs the assistance of a helicase and a SSB protein [1]. We tested whether or not a common origin of the genes putatively necessary for type II EcDNA replication, Rep and SSB, was supported by congruence in their phylogenies. The phylogeny of the SSB protein obtained for type II EcDNA was not congruent with that of the *Rep *of type II EcDNA, but rather with that of the *rep *of type I EcDNA: as shown in Figure 5a, the SSB proteins of both type I and type II EcDNAs are similar and related to the orthologous proteins of bacteria belonging to the low GC branch of Gram positives. Moreover the phylogeny of the SSB coding gene in phytoplasmal EcDNAs is similar to that of the 16S rDNA of phytoplasmas (Figure 5b). Most other ORFs borne by phytoplasmal EcDNAs can also be phylogenetically tracked to Gram positive bacteria and are highly similar between type I and type II EcDNAs. Figure 6 illustrates the composition of four EcDNAs, two of type I and two of type II, that are the complete EcDNA set of "*Ca*. P. asteris" strain AYWB. Each EcDNA encodes ORFs that are highly similar to their homologs in all other EcDNAs, except for those encoding the replication associated proteins; in fact the EcDNAs AYWB-pI and AYWB-pIII encode *Rep*, while AYWB-pII and AYWB-pIV encode *rep*. In synthesis, the phylogenetic analysis of SSB and the comparisons reported in figure 6 show that the phytoplasmal EcDNAs are strictly related replicons that share among each other sequences typical of Gram positive bacteria, while type II EcDNA have a replication associated protein that is not typical of Gram positive bacteria. As DNA regions with conflicting phylogenetic signals reflect incongruent genes histories due to recombination [26], this observation suggests that type II EcDNAs acquired a *Rep *gene through recombination. We then compared the tetranucleotide patterns used in the genes *rep *and *Rep *with those of the other coding sequences in the four EcDNAs of "*Ca*. P. asteris" strain AYWB. According to the results shown in figure 7 there is no correlation between the teranucleotide patterns used in *Rep *and the rest of the DNA sequences of the type I or type II EcDNAs, confirming that *Rep *did not co-evolve with the rest of the EcDNA replicons, including *rep*. Thus, according to the gene organization and nucleotide patterns, type II EcDNAs appear to be plasmids that have lost their *rep *and acquired an unrelated *Rep*, as a likely gain through horizontal gene transfer. The high level of sequence conservation shared by ORFs of type I and type II EcDNAs suggests that this gain was a relatively recent event.

In conclusion, evidences from replication associated protein similarity and EcDNA gene organization and composition show that the sequence similarity between the *Rep *genes of geminiviruses and phytoplasmas do not link geminiviruses to RCR plasmids of Gram positive bacteria, rather they indicate the existence in phytoplasmas of recombinant replicons containing a *Rep *with a different phylogenetic history from their host bacteria, presumably horizontally acquired from geminiviruses, i.e. viruses that share the same niche of phytoplasmas being insect transmitted and inhabiting the plant phloem.

### The elusive donor of the coat protein genes

In an attempt to define the origin of the geminivirus capsid, Krupovic and coworkers [12] hypothesized that phytoplasmal "plasmids" released upon lysis of the bacterial cell in the cytoplasm of the host plant cell obtained a coat protein (CP) coding gene from an unknown plant virus. Through modeling of the geminiviral CP Krupovic and coworkers [12] found that it fits the eight-stranded β-barrel folding model, like all isometric ssRNA plant viruses and several DNA viruses. Among viruses for which a 3D structure is available, the *Satellite tobacco necrosis virus *(STNV) was found, with a significant score, to be a suitable template for structural modeling of geminiviral CPs, as was also earlier reported in [27,28]. Krupovic and coworkers [12] constructed 3D models of geminiviral CPs and tested the stereochemical quality along with the X-ray structure of the STNV CP. In addition, they found similarity in the primary amino acid sequence between geminiviruses and STNV in a structure-based sequence alignment. On this basis they hypothesized that a phytoplasma "plasmid" may have recruited, through RNA/DNA recombination, the genetic information of a capsid protein from an icosahedral ssRNA virus similar to STNV resulting in the development of virions composed of two incomplete icosahedra large enough to accommodate its genome.

In assessing the strength of this hypothesis, it is important to stress that the virus capsid not only has the role of accommodating the viral genome, but also determines characteristics of transmission and infection of the virion. The *Geminiviridae *family is subdivided into four genera on the basis of their infection and genome characteristics [29]. Mastreviruses are transmitted by leafhoppers and have a single monopartite genome component. Members of the genus *Mastrevirus *have been found only in Europe, Africa, Asia and Australia where they infect monocots. Also Curtoviruses have monopartite genomes and are transmitted by leafhoppers, but they infect dicot plants. Begomoviruses, including the vast majority of geminiviruses, are transmitted by whiteflies, infect only dicots, and include species with a bipartite or a monopartite genome. The fourth genus, *Topocuvirus*, contains a single monopartite virus transmitted by treehoppers and appears to be a relatively recent result of a recombination between mastreviruses and begomoviruses [30]. The coat protein of geminiviruses is a determinant of vector transmission by either whiteflies or leafhoppers [31]. It has been shown by mutational analysis that the ability to be transmitted is determined by characteristics of the virion capsid [32,33]. In the hypothesis of Krupovic and coworkers [12], a parsimonious scenario should consider as suitable CP gene donor candidates viruses that not only have the same shape, but also share the same niche and confer similar transmission characteristics. It is relevant to mention that geminiviruses genome replicates in the nucleus (as it would a putative DNA plasmid ancestor), while most plant RNA viruses (including STNV) only invade the cytoplasm; the presence of the putative CP donor virus in a different cellular compartment would not favor genome recombination, and particularly the rare DNA-RNA recombination events. With regard to infection characteristics, CP donor candidates could be leafhopper- or whitefly- transmitted phloem-inhabiting viruses. However, as illustrated in Table 1, none of the known RNA virus families with members transmitted by leafhoppers or whiteflies share the structural characteristics of geminivirus, an issue that was taken as an indication of relatedness of their CPs by Krupovic and coworkers [12]. Viruses of the genera *Marafivirus *and *Waikavirus *have round isometric virions of about 30 nm, but with a T = 3 symmetry, which implies different protein-protein interactions than those occurring in virions with T = 1 symmetry, such as geminiviruses. In fact, our attempts to use these CPs as templates for structural modeling of the geminivirus CPs did not produce significant scores, according to the Structure Prediction MetaServer [34] (not shown). Moreover, although *Marafivirus *and *Waikavirus *are leafhopper transmitted they do not share the protein motif highly conserved in Mastrevirus that was shown to be relevant for transmission [28], suggesting that the ability of mastreviruses to be transmitted by leafhoppers has evolved independently from that of *Marafivirus *and *Waikavirus*.

**Table 1:** Virion characteristics of virus families including at least one species transmitted by leafhoppers or whiteflies

Virus Family or genus	Shape	Size	CP fold	Symmetry
Caulimoviridae (Badnavirus)	bacilliform	60-900 × 24-35 nm		
Closteroviridae	Filamentous	1000-2000 × 10-13		
Rhabdoviridae	Bullet-shaped or bacilliform	130-350 × 45-100		
Potyviridae	Filamentous	300-900 × 12-15		
Tenuivirus	helic al	3-10 nm × 950-1350		
Reoviridae	round	60-80 nm		T = 2
Tymoviridae (Marafivirus)	round	30 nm	Jelly-roll	T = 3
Secoviridae (Waikavirus)	round	25-30 nm	Jelly-roll	T = 3
Geminiviridae	geminate	30 × 18-20 nm	Jelly-roll	T = 1

Data derived from reference [51]

With no suitable donor candidates among the known leafhopper-or whitefly-transmitted viruses, a less parsimonious scenario has to be postulated to accommodate the hypothesis of Krupovic and coworkers [12]: the recruited CP gene conferred transmission characteristics that were different from those of geminiviruses, but in a later time a virus line evolved with infection characteristics and a niche that were, by pure chance, similar to those of the original donors of the *Rep *gene, i.e. the leafhopper-transmitted and phloem inhabiting phytoplasmas. This scenario would fit with STNV, that was indicated by Krupovic and coworkers [12] as the most closely related virus acting as a potential ancestor donor of capsid genes. However, if STNV, a virus transmitted by a fungus, was a donor of CP to the nascent geminivirus, then ssDNA viruses with a replication associated protein similar to geminivirus Rep but with transmission characteristics different from those of the present geminiviruses should have formed, a notion that contrasts with the present knowledge of plant virus diversity.

Despite the great diversity of known plant viruses, a non-geminivirus with Rep-like replication associated protein has never been found. Therefore, the less parsimonious version of the hypothesis of Krupovic and coworkers implies a *Geminiviridae *ancestral virus taxon that disappeared leaving no trace. On a contrasting line of evidence, a recently discovered geminivirus-related DNA mycovirus from the fungus *Sclerotinia sclerotiorum *(named SsHADV-1) [35] greatly differs in its CP from those of geminiviruses and from that of STNV as well. Here, we question that a poorly parsimonious hypothesis that also implies unlikely RNA/DNA recombination could be accepted. Indeed, data obtained from recent metagenomic studies suggest alternative hypothesis.

We conducted a BLASTP search in the EMBL sequence database for similarity to geminivirus CPs excluding the family *Geminiviridae*. We retrieved a protein encoded by a viral genome reconstructed by Rosario and coworkers [36] through data-mining of public viral metagenomes of reclaimed water (accession C6GIH8) that showed 29% identity and 39% similarity with the coat protein of the begomovirus *Crotalaria juncea virus *(accession A1EBG8). Recent metagenomic studies provide evidence of the existence of previously unknown viral genera [36-38]; some of these novel viral genomes similar to ssDNA circoviruses (a group of animal viruses) were found to have predicted CPs different from known circovirus and more similar to geminivirus CPs [36]. Searching the sequences derived from marine environment metagenomic studies in datasets available from the community cyberinfrastructure for advanced marine microbial ecology research and analysis (CAMERA, website http://camera.calit2.net) by BLASTP we found several sequences of likely viral origin that showed significant similarity to geminivirus CPs. Table 2 shows that the similarity of some of these entries retrieved with selected *Geminiviridae *CPs are comparable with those calculated between CPs of begomoviruses and mastreviruses (that range from 16 to 27% identity and 27 to 46% similarity). According to Table 2, there are sequences from marine environments that appear to be better candidates than STNV for being putative relatives of geminivirus CPs. Although it cannot be excluded that such viruses are derivative rather than ancestors of geminiviruses, our analysis show that geminivirus ancestors could have evolved their CP in marine environments before their adaptation as pathogens of land plants, and therefore their origin could be explained without having recourse to unlikely and poorly parsimonious scenarios.

**Table 2:** Amino acid similarities and identities of some protein sequences deduced from entries of metagenomic study with selected geminivirus CPs

					Alignment
Query sequence	Most similar geminivirus CP		Identity	Similarity	length
JCVIPEP1105104354663	Q5UAE8	Indian cassava mosaic virus	23.4	39.7	250
JCVIPEP1105107973141	A7UGA6	Sweet potato leaf curl virus	22.4	36.6	183
JCVIPEP1105096364985	A7UGA6	Sweet potato leaf curl virus	22.8	37.6	250
JCVIPEP1105092295201	C7TPA7	Mung bean yellow mosaic India virus	27.3	44.6	139
JCVIPEP1105092294601	Q645H3	Corchorus yellow vein virus	18.5	32.4	222
JCVIPEP1105092292339	Q19LG2	Tomato golden mottle virus	27.6	42.9	217
SNTV (PDBID:2BUK)	Q4LAS1	Wheat dwarf virus	18.7	32.2	267

The amino acid similarities and identities of the SNTV CP is also included for comparison.

In conclusion, although the origin of the geminivirus CP cannot be determined with certainty, the origin from a ssRNA virus such as SNTV appears to be unlikely compared to other hypotheses on the basis of similarity analysis, the absence of any remnant of a non-leafhopper/whitefly-transmitted plant virus encoding Rep, and the requirement of a DNA/RNA recombination event in incongruent cell compartments.

Given the evidence of a distant relationship between the CPs of geminiviruses and STNV, a common origin for both spherical and geminate virions with T = 1 icosahedral symmetry remains an interesting hypothesis; the information reported here only shows that the idea that the evolution from the common ancestor to the present virions occurred in land plants is not sufficiently supported. Several lines of evidence further indicate that geminiviruses evolved earlier, from remote ancestors existing 450 million years ago [39], and there is molecular evidence that begomoviruses and mastreviruses were already differentiated at the time of the Gondwana separation [40], i.e. before the phytoplasma phylogenetic branch arose from the insect colonizing AAP (Acholeplasma - Anaeroplasma - Phytoplasma) lineage of Mollicutes (estimated as 180 million years in [41]). This course of evolutionary events is also compatible with a common origin of ssDNA viruses of plants, in agreement with the results gathered by Gibbs and Weiler [42] who detected several traits in common between geminiviruses and nanoviruses strongly suggesting their common origin, a notion consistent with both the transmission characteristics and type of replication.

It is tempting to conclude that the apparent evolutionary isolation of geminiviruses deduced by the analysis of RCR replicons in plants is only due to the limitation of our narrow view on life diversity.

### Filling the gaps: a hypothesis on the origin and success of phytoplasmal type II EcDNA

Our results from sequence data analysis are consistent with a recombination event between phytoplasma plasmids (type I EcDNAs) and the geminivirus genome giving rise to type II EcDNAs in phytoplasmas. Krupovic and coworkers [12] have discarded this hypothesis because geminiviruses "maintained features of prokaryotic replicons, such as typical bacterial promoter sequences" and "are in some instances still able to replicate their DNA in bacterial cells". It may be useful to stress that a remote bacterial origin is definitely not in contrast with a hypothesis of a more recent recombination event. There are also reasons to question the putative origin of geminivirus *Rep *from bacterial plasmids. Kapitonov and Jurka [1] suggested that geminiviruses might have evolved from plant RC transposons rather than from prokaryotic RC replicons. Plant RC transposons (helitrons) encode their own helicase and SSB. Moreover, some geminiviruses can replicate in the Gram negative *Agrobacterium tumefaciens *[43], while, to our knowledge, no RCR plasmid of the pLS1 family has been reported to replicate in Gram negatives. In addition, there is no evidence that geminivirus Rep is functional in a bacterial background that support replication of RCR plasmids. We have tested the ability of different constructs containing phytoplasmal *Rep *to replicate in *Bacillus subtilis*. We inserted the entire NJAY EcDNA into pJM103 (a pUC18 derivative that can replicate in *E. coli *but not in *B. subtilis *and contains a chloramphenicol resistance that is expressed in *B. subtilis *[44]), but found no evidence of replication of the construct in *B. subtilis *(results not shown). Thus, the replication in *A. tumefaciens *does not appear to be strong evidence of a geminivirus relationship with RCR plasmids.

The sequence of the complete genome of several phytoplasmas showed that these organisms have incomplete nucleotide synthesis pathways and therefore depend on their host for nucleotides [8,45,46]. No transport system for nucleosides or nucleotides has been identified yet in the phytoplasma genomes, and, since no information on how they obtain the necessary nucleotides for replication is available, uptake and recycling of nucleic acids from the host plant may play a prominent role. It has also been shown that phytoplasmas have a highly active recombination system. Indeed, sequences similar to truncated geminivirus *Rep *have been found in the chromosome of several phytoplasmas. Thus, geminivirus DNA in the phloem may have been readily available for internalization and incorporation into the phytoplasma chromosomal or extrachromosomal DNA by recombination.

Once acquired by recombination, the survival and sequence conservation [3] of *Rep *in phytoplasmas may derive from its contribution to the propagation and spread of plasmid borne functions. Namba and coworkers [47] have highlighted the possible implication of the phytoplasma plasmid borne ORF3 in determining insect transmissibility and showed that a non-insect-trasmissible variant of the same phytoplasma strains lacked ORF3. Thus, a plasmid encoded sequence may have a relevant role in phytoplasma epidemiology.

According to our Southern blot analyses (not shown) and other studies [46] no EcDNA was detected in phytoplasmas such as "*Ca*. P. mali", "*Ca*. P. pyri", "*Ca*. P. vitis", "*Ca*. P. prunorum" that are monophagous and have a narrow insect vector range. Conversely EcDNAs have been reported in strains of the polyphagous species "*Ca*. P. asteris", "*Ca*. P. australiense", "*Ca*. P. pruni" and "*Ca*. P. trifolii", that are transmitted by a wider range of insect vector species [3,5-9]. There are several reports over the last 15 years of molecular analysis of phytoplasma diversity that indicate that the infection by two or more polyphagous phytoplasmas is a common event in herbaceous plants; besides, transmission of phytoplasma strains by different insect species has been found to be the basis of epidemics and outbreaks of new diseases [48]. In this context, an EcDNA carrying ORF3 and propagating among polyphagous phytoplasmas possibly contributed to widen the insect vector range. Our analysis of the untranslated region of NJAY phytoplasma EcDNA revealed that it includes a remnant of ORF3 (figure 4). Since NJAY phytoplasma EcDNA, like several other EcDNA sequences in the database, has been obtained from a phytoplasma strain isolated in an experimental host and propagated for many years by graft transmission rather than insect vectoring, the NJAY EcDNA could have initiated a process of reductive evolution, as recently reported [49], loosing a functional ORF3. A search among other phytoplasmal EcDNA sequences revealed that functional or incomplete ORF3 homologs are present in 19 out of the 30 EcDNAs fully sequenced so far.

The potential contribution in broadening insect vector specificity by propagating ORF3 horizontally among phytoplasmas may be the cause of the conservation of EcDNAs, including type II EcDNAs that may have originated by recombination. Although a search for the canonical nonanucleotide sequence in the untranslated region of NJAY type II EcDNA was unsuccessful, we detected a variant with 8 conserved nts (not shown); the recent report that high-affinity Rep-binding is not required for the replication of a geminivirus DNA [50] gives ground to the hypothesis that, upon recombination, a geminivirus Rep may have functionally substituted rep in catalyzing the replication of DNA sequences, representing a selective advantage for the host organism. We may speculate that the propagation and spread of ORF3 may have granted conservation of both EcDNA types.

Since phytoplasmas belonging to some phylogenetic clades do not have remnants of *Rep *that are conversely common in other strains, the phytoplasma type II EcDNA should have appeared after the separation of the major phytoplasma clades, well after the appearance on earth of vascular plants and probably the origin of geminiviruses.

## Conclusion

The data presented here explain the origin of phytoplasmal type II EcDNAs and support the rejection of the hypothesis that geminiviruses evolved from phytoplasma plasmids, even though the evolutionary history of geminiviruses remains to be clarified. Nevertheless, in agreement with recent reviews on this topic [39], a more in depth investigations of environments different from higher plants is expected to provide sound answers.

## List of abbreviations

Rep: viral replication associated protein; rep: bacterial replication associated protein; ssDNA: single stranded DNA; EcDNA: extrachromosomal DNA.

## Authors' contributions

FS carried out the amplification, cloning and sequencing of the phytoplasma plasmid, carried out the phylogenetic analyses, prepared the figures and tables and helped with writing the manuscript. EC contributed to data analysis. SP carried out DNA analysis by southern blot and contributed to cloning and manuscript writing. EN contributed to data mining and manuscript writing. GF conceived the study and its design, coordinated the work and wrote the manuscript. All authors read and approved the final manuscript.

## Supplementary Material

Additional file 1 **Supplementary Table 1**. Designation, related species, disease caused, origin and 16Sr group affiliation of phytoplasmas screened for EcDNAs by Southern blot. Click here for file

Additional file 2 **Supplementary Table 2**. Geminivirus, phytoplasmal and bacterial sequences reported in figures 1-5. Click here for file

Additional file 3 **Supplementary Table 3**. Oligonucleotide primers used for EcDNA NJAY detection and sequencing. Click here for file

## References

[B1] Kapitonov VV Jurka J Rolling-circle transposons in eukaryotes Proc Natl Acad Sci USA 2001 98 8714 8719 10.1073/pnas.151269298 11447285 PMC37501

[B2] Koonin E Ilyina T Geminivirus replication proteins are related to prokaryotic plasmid rolling circle DNA replication initiator J Gen Virol 1992 10 2763 2766 10.1099/0022-1317-73-10-2763 1402808

[B3] Rekab D Carraro L Schneider B Seemüller E Chen JC Chang CJ Locci R Firrao G Geminivirus-related extrachromosomal DNAs of the X-clade phytoplasmas share high sequence similarity Microbiology 1999 145 1453 1459 10.1099/13500872-145-6-1453 10411272

[B4] Nishigawa H Miyata S Oshima K Sawayanagi T Komoto A Kuboyama T Matsuda I Tsuchizaki T Namba S In planta expression of a protein encoded by the extrachromosomal DNA of a phytoplasma and related to geminivirus replication proteins Microbiology 2001 147 507 513 11158368 10.1099/00221287-147-2-507

[B5] Nishigawa H Oshima K Kakizawa S Jung H Kuboyama T Miyata S Ugaki M Namba S Evidence of intermolecular recombination between extrachromosomal DNAs in phytoplasma: a trigger for the biological diversity of phytoplasma? Microbiology 2002 148 1389 1396 11988512 10.1099/00221287-148-5-1389

[B6] Liefting LW Andersen MT Lough TJ Beever RE Comparative analysis of the plasmids from two isolates of "Candidatus Phytoplasma australiense" Plasmid 2006 56 138 144 10.1016/j.plasmid.2006.02.001 16620976

[B7] Liefting LW Shaw ME Kirkpatrick BC Sequence analysis of two plasmids from the phytoplasma beet leafhopper-transmitted virescence agent Microbiology 2004 150 1809 1817 10.1099/mic.0.26806-0 15184567

[B8] Bai X Zhang J Ewing A Miller SA Jancso Radek A Shevchenko DV Tsukerman K Walunas T Lapidus A Campbell JW Hogenhout SA Living with genome instability: the adaptation of phytoplasmas to diverse environments of their insect and plant hosts J Bacteriol 2006 188 3682 3696 10.1128/JB.188.10.3682-3696.2006 16672622 PMC1482866

[B9] Tran-Nguyen LT Gibb KS Extrachromosomal DNA isolated from tomato big bud and Candidatus Phytoplasma australiense phytoplasma strains Plasmid 2006 56 153 166 16879868 10.1016/j.plasmid.2006.05.009

[B10] Namba S Oshima K Gibb KS Blanchard A, Browning G Phytoplasma genomics Mycoplasmas: Molecular Biology, Pathogenicity and Strategies for Control 2005 Norfolk, U.K., Horizon Bioscience 97 133

[B11] Firrao G Garcia-Chapa M Marzachì C Phytoplasmas: genetics, diagnosis and relationships with the plant and insect host Front Biosci 2007 12 1353 1375 10.2741/2153 17127387

[B12] Krupovic M Ravantti JJ Bamford DH Geminiviruses: a tale of a plasmid becoming a virus BMC Evol Biol 2009 9 112 10.1186/1471-2148-9-112 19460138 PMC2702318

[B13] Ahrens U Seemüller E Detection of DNA of plant pathogenic mycoplasma-like organisms by a polymerase chain reaction that amplifies a sequence of the 16S rRNA gene Phytopathology 1992 82 828 832 10.1094/Phyto-82-828

[B14] Tamura K Dudley J Nei M Kumar S Molecular Evolutionary Genetics Analysis (MEGA) software version 4.0 Mol Biol Evol 2007 24 1596 1599 10.1093/molbev/msm092 17488738

[B15] Felsenstein J PHYLIP (Phylogeny Inference Package) version 3.6 2010 Washington University, Genome Sciences Department Distributed by the author

[B16] Rice P Longden I Bleasby A EMBOSS: the European Molecular Biology Open Software Suite Trends Genet 2000 16 276 277 10.1016/S0168-9525(00)02024-2 10827456

[B17] R Development Core Team R: a Language and Environment for Statistical Computing 2007 Vienna, R Foundation for Statistical Computing http://www.R-project.org

[B18] Huelsenbeck JP Hillis DM Nielsen R A likelihood-ratio test of monophyly Syst Biol 1996 45 546 558 10.1093/sysbio/45.4.546

[B19] Schliep KP Phangorn: phylogenetic analysis in R Bioinformatic 2011 27 592 593 10.1093/bioinformatics/btq706 PMC3035803 21169378

[B20] Strope CL Abel K Scott SD Moriyama EN Biological sequence simulation for testing complex evolutionary hypotheses: indel-Seq-Gen version 2.0 Mol Biol Evol 2009 26 2581 2593 10.1093/molbev/msp174 19651852 PMC2760465

[B21] Teeling H Waldmann J Lombardot T Bauer M Glöckner FO TETRA: a web-service and a stand-alone program for the analysis and comparison of tetranucleotide usage patterns in DNA sequences BMC Bioinf 2004 5 163 170 10.1186/1471-2105-5-163 PMC529438 15507136

[B22] Bergemann AD Whitley JC Finch LR Homology of mycoplasma plasmid pADB201 and staphylococcal plasmid pE194 J Bacteriol 1989 171 593 595 2644210 10.1128/jb.171.1.593-595.1989 PMC209630

[B23] Oshima K Kakizawa S Nishigawa H Kuboyama T Miyata S Ugaki M Namba S A plasmid of phytoplasma encodes a unique replication protein having both plasmid- and virus-like domains: clue to viral ancestry or result of virus/plasmid recombination? Virology 2001 285 270 277 10.1006/viro.2001.0938 11437661

[B24] Gibbs MJ Smeianov VV Steele JL Upcroft P Efimov Ba Two families of rep-like genes that probably originated by interspecies recombination are represented in viral, plasmid, bacterial, and parasitic protozoan genomes Mol Biol Evol 2006 23 1097 1100 10.1093/molbev/msj122 16531508

[B25] Kuboyama T Huang CC Lu X Sawayanagi T Kanazawa T Kagami T Matsuda I Tsuchizaki T Namba S A plasmid isolated from phytopathogenic onion yellows phytoplasma and its heterogeneity in the pathogenic phytoplasma mutant Mol Plant Microbe Interact 1998 11 1031 1037 10.1094/MPMI.1998.11.11.1031 9805390

[B26] Lawrence JG Retchless AC The myth of bacterial species and speciation Biology and Philosophy 2010 25 569 588 10.1007/s10539-010-9215-5

[B27] Zhang W Olson N Baker T Faulkner L Agbandje-McKenna M Boulton M Davies J McKenna R Structure of the Maize streak virus geminate particle Virology 2001 279 471 477 10.1006/viro.2000.0739 11162803

[B28] Bottcher B Unseld S Ceulemans H Russel R Jeske H Geminate Structures of African Cassava Mosaic Virus J Virol 2004 78 6758 6765 10.1128/JVI.78.13.6758-6765.2004 15194750 PMC421685

[B29] Rybicki EB Briddon RW Brown JK Fauquet CM Maxwell DP Harrison BD Markham PG Bisaro DM Robinson D Stanley J Regenmortel MHV, Fauquet CM, Bishop DHL, Carstens EB, Estes MK, Lemon SM, Maniloff J, Mayo MA, McGeoch DJ, Pringle CR, Wickner RB Family Geminiviridae Virus Taxonomy Seventh Report of the International Committee on Taxonomy of Viruses 2000 San Diego: Academic Press 285 297

[B30] Rojas MR Hagen C Lucas WJ Gilbertson RL Exploiting chinks in the plant's armor: evolution and emergence of geminiviruses Annu Rev Phytopathol 2005 43 361 394 10.1146/annurev.phyto.43.040204.135939 16078889

[B31] Briddon R Pinner M Stanley J Markham P Geminivirus coat protein gene replacement alters insect specificity Virology 1990 177 85 94 10.1016/0042-6822(90)90462-Z 2353465

[B32] Noris E Vaira A Caciagli P Masenga V Gronenborm B Accotto G Amino acids in the capsid protein of Tomato yellow leaf curl virus that are crucial for systemic infection, particle formation, and insect transmission J Virol 1998 72 10050 10057 9811744 10.1128/jvi.72.12.10050-10057.1998 PMC110531

[B33] Caciagli P Medina Piles V Marian D Vecchiati M Masenga V Mason G Falcioni T Noris E Virion stability is important for the circulative transmission of tomato yellow leaf curl sardinia virus by Bemisia tabaci, but virion access to salivary glands does not guarantee transmissibility J Virol 2009 83 5784 5795 10.1128/JVI.02267-08 19321611 PMC2681986

[B34] Ginalski K Elofsson A Fischer D Rychlewski L 3D-Jury: a simple approach to improve protein structure predictions Bioinformatics 2003 19 1015 1018 10.1093/bioinformatics/btg124 12761065

[B35] Yu X Li B Fu Y Jiang D Ghabrial S Li G Peng Y Xie J Cheng J Huang J Yi X A geminivirus-related DNA mycovirus that confers hypovirulence to a plant pathogenic fungus Proc Natl Acad Sci USA 2010 107 8387 8392 10.1073/pnas.0913535107 20404139 PMC2889581

[B36] Rosario K Duffy S Breitbart M Diverse circovirus-like genome architectures revealed by environmental metagenomics J Gen Virol 2009 90 2418 2424 10.1099/vir.0.012955-0 19570956

[B37] Kim KH Chang HW Nam YD Roh SW Kim MS Sung Y Jeon CO Oh HM Bae JW Amplification of uncultured viruses from rice paddy soil Appl Environ Microbiol 2008 74 5975 5985 10.1128/AEM.01275-08 18708511 PMC2565953

[B38] Ng TF Manire C Borrowman K Langer T Ehrhart L Breitbart M Discovery of a novel single-stranded DNA virus from a sea turtle fibropapilloma by using viral metagenomics J Virol 2009 83 2500 2509 10.1128/JVI.01946-08 19116258 PMC2648252

[B39] Nawaz-Ul-Rehman MS Fauquet CM Evolution of geminiviruses and their satellites FEBS Lett 2009 583 1825 1832 10.1016/j.febslet.2009.05.045 19497325

[B40] Ha C Coombs S Revill P Harding R Vu M Dale J Molecular characterization of begomoviruses and DNA satellites from Vietnam: additional evidence that the New World geminiviruses were present in the Old World prior to continental separation J Gen Virol 2008 89 312 326 10.1099/vir.0.83236-0 18089756

[B41] Maniloff J Reconstructing the timing and selective events of mycoplasma evolution Abstracts of the 13th International Congress of the International Organization for Mycoplasmology (IOM): 14-19 July 2000 2000 Fukuoka (JP) 65

[B42] Gibbs MJ Weiller GF Evidence that a plant virus switched hosts to infect a vertebrate and then recombined with a vertebrate-infecting virus Proc Natl Acad Sci USA 1999 96 8022 8027 10.1073/pnas.96.14.8022 10393941 PMC22181

[B43] Selth LA Randles JW Rezaian MA Agrobacterium tumefaciens supports DNA replication of diverse geminivirus types FEBS Lett 2002 516 179 182 10.1016/S0014-5793(02)02539-5 11959128

[B44] Perego M Hoch JA Negative regulation of Bacillus subtilis sporulation by the spo0E gene product J Bact 1991 173 2514 2250 1901567 10.1128/jb.173.8.2514-2520.1991 PMC207815

[B45] Oshima K Kakizawa S Nishigawa H Jung H Wei W Suzuki S Arashida R Nakata D Miyata S Ugaki M Namba S Reductive evolution suggested from the complete genome sequence of a plant-pathogenic phytoplasma Nat Genet 2004 36 27 29 10.1038/ng1277 14661021

[B46] Kube M Schneider B Kuhl H Dandekar T Heitmann K Migdoll AM Reinhardt R Seemüller E The linear chromosome of the plant-pathogenic mycoplasma '*Candidatus *Phytoplasma mali' BMC Genomics 2008 9 306 10.1186/1471-2164-9-306 18582369 PMC2459194

[B47] Ishii Y Kakizawa S Hoshi A Maejima K Kagiwada S Yamaji Y Oshima K Namba S In the non-insect-transmissible line of onion yellows phytoplasma (OY-NIM), the plasmid-encoded transmembrane protein ORF3 lacks the major promoter region Microbiology 2009 155 2058 2067 10.1099/mic.0.027409-0 19372166

[B48] Lee IM Gundersen-Rindal DE Bertaccini A Phytoplasma: ecology and genomic diversity Phytopathology 1998 88 1359 1366 10.1094/PHYTO.1998.88.12.1359 18944840

[B49] Ishii Y Oshima K Kakizawa S Hoshi A Maejima K Kagiwada S Yamaji Y Namba S Process of reductive evolution during 10 years in plasmids of a non-insect-transmissible phytoplasma Gene 2009 446 51 57 10.1016/j.gene.2009.07.010 19631261

[B50] Lin B Akbar Behjatnia SA Dry IB Randles JW Rezaian MA High-affinity Rep-binding is not required for the replication of a geminivirus DNA and its satellite Virology 2003 305 353 363 10.1006/viro.2002.1671 12573580

[B51] Fuchs M Transmission specificity of plant viruses by vectors J Plant Pathol 2005 87 153 165

[B52] Park M Kim M Lee K Hwang S Ahn TI Characterization of a cryptic plasmid from an alpha-proteobacterial endosymbiont of Amoeba proteus Plasmid 2009 61 78 87 10.1016/j.plasmid.2008.09.007 18951917

