# Supplementary material for: On the alleged origin of geminiviruses from extrachromosomal DNAs of phytoplasmas
Source: BMC Evol Biol. 2011 Jun 28;11:185. doi: 10.1186/1471-2148-11-185 (PMC3154185; doi:10.1186/1471-2148-11-185)
Supplement: Additional file 1 — Supplementary Table 1. Designation, related species, disease caused, origin and 16Sr group affiliation of phytoplasmas screened for EcDNAs by Southern blot. [file 1471-2148-11-185-S1.DOC]

| **Strain** | **Species** | **Associated disease** | **Origin** | **16Sr classification** |
| --- | --- | --- | --- | --- |
| NJAY | ‘*Candidatus* Phytoplasma asteris’ | New Jersey aster yellows | USA | I-A |
| AY | ‘*Ca.* P. asteris’ | Aster yellows | Germany | I-B |
| TWB | ‘*Ca.* P. asteris’ | Tagete witches’-broom | Italy | I-B |
| CA | ‘*Ca.* P. asteris’ | Aster yellows | Italy | I-C |
| LEO | ‘*Ca.* P. asteris’ | Aster yellows | Italy | I-C |
| CPhy | ‘*Ca.* P. asteris’ | Clover phyllody | Canada | I-C |
| TBB | ‘*Ca*. P. aurantifolia’ | Tomato big bud | Australia | II-D |
| FABA | ‘*Ca*. P. aurantifolia’ | Faba bean phyllody | USA | II-C |
| LN-I | ‘*Ca*. P. pruni’ | Leafhopper isolate | Italy | III-B |
| VAC | ‘*Ca*. P. pruni’ | *Vaccinium* witches’ broom | Germany | III-F |
| JR (=PoiBi) | ‘*Ca*. P. pruni’ | Poinsettia branch inducine | USA | III-H |
| FD | Not described | Flavescence dorée | France | V-A |
| EY | ‘*Ca.* P. ulmi | Elm yellows | USA | V-A |
| PWB | *'Ca*. P. trifolii’ | Potato witches’-broom | USA | VI-A |
| BLTVA | *'Ca*. P. trifolii’ | Beat leafhopper-transmitted virescence agent | USA | VI-A |
| AP15 | ‘*Ca*. P. mali’ | Apple proliferation | Germany | X-A |
| LNS1 | ‘*Ca*. P. prunorum’ | European stone fruit yellow | Italy | X-B |
| PD | ‘*Ca.* P. pyri’ | Pear decline | Germany | X-C |
| A-SLO | Not described | Stolbur | Slovenia | XII-A |
| P-TV | Not described | Stolbur | Italy | XII-A |
| BA | Not described | Stolbur | Italy | XII-A |
| SE | Not described | Stolbur | Italy | XII-A |
| T256 | Not described | Stolbur | Italy | XII-A |
