# Supplementary material for: On the alleged origin of geminiviruses from extrachromosomal DNAs of phytoplasmas
Source: BMC Evol Biol. 2011 Jun 28;11:185. doi: 10.1186/1471-2148-11-185 (PMC3154185; doi:10.1186/1471-2148-11-185)
Supplement: Additional file 2 — Supplementary Table 2. Geminivirus, phytoplasmal and bacterial sequences reported in figures 1-5. [file 1471-2148-11-185-S2.DOC]

| **STRAIN** | **ABBREVIATION** | **ACCESSION N° REP, SSB, 16S rRNA** |
| --- | --- | --- |
| Porcine circovirus | CIR1 | Q3Y8L6_PCV2 (UniProt) |
| Duck circovirus | CIR2 | A7LI82_9CIRC (UniProt) |
| Pigeon circovirus | CIR3 | Q91G98_PICV (UniProt) |
| Gull circovirus | CIR4 | [DQ845074](http://www.ebi.ac.uk/cgi-bin/expasyfetch?DQ845074) (EMBL) |
| Canary circovirus | CIR5 | [AJ301633](http://www.ebi.ac.uk/cgi-bin/expasyfetch?AJ301633) (EMBL) |
| Beak and father disease virus | CIR6 | NP_047275 (GenBank) |
| Blainvillea yellow spot virus | B1 | NC_004042 (GenomeMine) |
| Kudzu mosaic virus | B2 | YP_001333680 (GenBank) |
| Macroptilum mosaic PuertoRico virus | B3 | NP_671461 (GenBank) |
| Tomato yellow leaf curl Sardinia virus | B4 | YP_459911 (GenBank) |
| Euphorbia mosaic virus | B5 | YP_717930 (GenBank) |
| Spilantes yellow vein virus | B6 | YP_00128576 (GenBank) |
| Emilia yellow vein virus | B7 | YP_001661461 (GenBank) |
| Beet curly top virus | C1 | NP_040557 (GenBank) |
| Beet mild curly top virus | C2 | Q65418_9GEMI (UniProt) |
| Sugarcane streak virus | M1 | REP_SSVN (UniProt) |
| Wheat dwarf virus | M2 | Q4LAR8_PEA (UniProt) |
| Panicum streak virus | M3 | REP_PASVK (UniProt) |
| Chloris striate mosaic virus | M4 | REP_CSMV (UniProt) |
| Miscanthus streak virus | M5 | REP_MISV9 (UniProt) |
| Tobacco dwarf virus | M6 | NP_620726 (GenBank) |
| EcPASb11 | TII.1 | YP_001965310 (GenBank) |
| EcPAPh2 | TII.2 | YP_001965305 (GenBank) |
| EcPaWBNy_1 | TII.3 | YP_001708784, EF426472 (GenBank) |
| EcOY | TII.4 | REPE_ONYPE (UniProt), NC_012088 (GenBank) |
| *Porphyra pulchra* | P.p | AF106328 (EMBL) |
| pOYW1 | TI.1 | [AB010421](http://www.ebi.ac.uk/cgi-bin/emblfetch?AB010421)**,** AB056859 (GenBank) |
| pCPa | TI.2 | Q1WM07_PHYAS (UniProt) |
| pAYWB-IV | TI.3 | Q2NID8_AYWB_P (UniProt), CP000065 (GenBank) |
| *Streptococcus agalactiae*_pLS1 | BA1 | NC_007432 (GenBank) |
| *Streptococcus equisimilis*_pSdyT132 | BA2 | Q0H7S3_STREQ (UniProt) |
| *Lactobacillus rhamnosus*_pLR001 | BA3 | B5RSE1_LACRH (UniProt) |
| *Lactococcus lactis*_pWV01 | BA4 | [X56954](http://www.ebi.ac.uk/cgi-bin/emblfetch?X56954) (GenBank) |
| *Mycoplasma capricolum*_pMCA0277 | BA5 | Q2SSK0_MYCCT (UniProt) |
| *Mycoplasma mycoides*_pKMK1 | BA6 | Q50243_MYCMY (UniProt) |
| *Mycoplasma leachii*_pBG7AU | BA7 | Q9F870_9MOLU (UniProt) |
| *Clostridium nexile*_plasmid | BA8 | B6FMZ0_9CLOT (UniProt) |
| *Bacillus cereus*_pE33L5 | BA9 | Q4V101_BACCZ (UniProt) |
| *Lactobacillus gasseri*_plasmid | BA10 | C4VTL7_9LACO (UniProt) |
| *Staphylococcus aureus*_pEI94 | BA11 | REPY_STAAU (UniProt) |
| *Bacillus thuringensis*_pGI1 | BA12 | NP_705751 (GenBank) |
| *Bacillus thuringensis*_pBMBt1 | BA13 | Q5PXQ4_BACUD (UniProt) |
| *Bacillus thuringensis*_pTX14-3 | BA14 | [X62876](http://www.ebi.ac.uk/cgi-bin/emblfetch?X62876) (GenBank) |
| *Bacillus mycoides*_pBMYdx | BA15 | Q9L3K9_BACMY (UniProt) |
| *Geobacillus stearotermophilus*_pSTK1 | BA16 | NC_002062 (GenBank) |
| *Bacillus thuringensis*_pTX14-1 | BA17 | [P71099](http://www.ebi.ac.uk/cgi-bin/emblfetch?U67921) (UniProt) |
| *Staphylococcus aureus*_pUB110 | BA18 | NP_040434 (GenBank) |
| *Bacillus sp._*pBAA1 | BA19 | REP_BACSP (UniProt) |
| *Staphylococcus aureus*_pBC16 | BA20 | NP_043526 (GenBank) |
| *Bacillus thuringensis*_pGI3 | BA21 | [Y11173](http://www.ebi.ac.uk/cgi-bin/emblfetch?Y11173) (GenBank) |
| *Lactobacillus plantarum*_plasmid | BA22 | COFYA5_9FIRM (UniProt) |
| *Staphylococcus aureus*_pC194 | BA23 | P03862 (UniProt) |
| *Corynebacterium diphteriae*_pEP2 | BA24 | Q52263 (UniProt) |
| *Brevibacterium flavum*_pGA1 | BA25 | X90817 (GenBank) |
| *Corynebacterium glutamicum*_pSR1 | BA26 | Q46071_CORGL (UniProt) |
| *Lactobacillus crispatus*_plasmid | BA27 | C2KH25_9LACO (UniProt) |
| *Leuconostoc mesenteroides*_plasmid | BA28 | C2KI24_LEUMC (UniProt) |
| *Lactobacillus acidophilus*_plasmid | BA29 | Q5FM55_LACAC (UniProt) |
| *Bifidobacterium catenulatum*_ pDSM16992 | BA30 | ZP_03324227 (GenBank) |
| Soil metagenome C112 | SO | ABQX01000001 (GenBank) |
| EcOY-M |  | AB076263 (GenBank) |
| pOY-M |  | NC_012089 (GenBank) |
| EcOY-NIM |  | NC_006903 (GenBank) |
| EcNJAY |  | this work |
| EcAYWB-I |  | CP000062 (GenBank) |
| pAYWB-II |  | CP000063 (GenBank) |
| EcAYWB-III |  | CP000064 (GenBank) |
| pJHW |  | AB064396 (GenBank) |
| EcPaWBNy-2 |  | EF426473 (GenBank) |
| ‘*Candidatus* Phytoplasma mali’ |  | YP_002004429, EF392656 (GenBank) |
| *‘Candidatus* Phytoplasma asteris’ |  | YP_456346.1, AY265213 (GenBank) |
| *Acholeplasma laidlawii* |  | YP_001620196, CP000896 (GenBank) |
| *Spiroplasma citri* |  | CAK99379, NC_007294 (GenBank) |
| *Bacillus subtilis* |  | NP_391970, GQ892930 (GenBank) |
| *Lactobacillus sakei* |  | YP_394621 M23727 (GenBank) |
| *Streptococcus pneumoniae* |  | CAR69285, M23730 (GenBank) |
| *Clostridium botulinum* |  | ACA44700 M23732 (GenBank) |
| *Pseudomonas aeruginosa* |  | AAP22521, CP000744 (GenBank) |
| *Salmonella enterica* |  | ACQ77892, NC_011149 (GenBank) |
| *Escherichia coli* |  | ACQ78066, FN554766 (GenBank) |
