# Supplementary material for: On the alleged origin of geminiviruses from extrachromosomal DNAs of phytoplasmas
Source: BMC Evol Biol. 2011 Jun 28;11:185. doi: 10.1186/1471-2148-11-185 (PMC3154185; doi:10.1186/1471-2148-11-185)
Supplement: Additional file 3 — Supplementary Table 3. Oligonucleotide primers used for EcDNA NJAY detection and sequencing. [file 1471-2148-11-185-S3.DOC]

| **Primer** | **Oligonucleotide sequence**  **5’->3’** | **Annealing temperature** | **Product size** |
| --- | --- | --- | --- |
| fAYWBrep | cmmartgtsmtttrggtaaag | 55°C | 500 bp |
| rAYWBrep | atmacaatdgatttaggt |  |  |
| fAYWBest | ggatgdwwwccdtcaakrtcaa | 50°C | 2000 bp |
| rAYWBest | gacctaaatchattgtkat |  |  |
| fNJCP | ccatcaatgtcaaagaatc | 53°C | 2000 bp |
| rNJCP | gatggcgacttcattgaag |  |  |
| rNJ1 | attgttgcagtcaatacac | 52°C | 800 bp |
| fNJa | acggtcataagtttcatga |  |  |

Additional file 3: Oligonucleotide primers used for EcDNA NJAY detection and sequencing (M:A/C, R:A/G, S:G/C, W:A/T, K:G/T, D:A/G/T, H:A/C/T).
